# Comparison of Airway Responses in Sheep of Different Age in Precision-Cut Lung Slices (PCLS)

**DOI:** 10.1371/journal.pone.0097610

**Published:** 2014-09-17

**Authors:** Verena A. Lambermont, Marco Schlepütz, Constanze Dassow, Peter König, Luc J. Zimmermann, Stefan Uhlig, Boris W. Kramer, Christian Martin

**Affiliations:** 1 Department of Pediatrics, Maastricht University Medical Center, Maastricht, the Netherlands; 2 Institute of Pharmacology and Toxicology, University Hospital Aachen, Aachen, Germany; 3 Institute of Anatomy, University of Lübeck, Airway Research Center North (ARCN), Member of the German Center for Lung Research (DZL), Lübeck, Germany; Institute of Lung Biology and Disease (iLBD), Helmholtz Zentrum München, Germany

## Abstract

**Background:**

Animal models should display important characteristics of the human disease. Sheep have been considered particularly useful to study allergic airway responses to common natural antigens causing human asthma. A rationale of this study was to establish a model of ovine precision-cut lung slices (PCLS) for the *in vitro* measurement of airway responses in newborn and adult animals. We hypothesized that differences in airway reactivity in sheep are present at different ages.

**Methods:**

Lambs were delivered spontaneously at term (147d) and adult sheep lived till 18 months. Viability of PCLS was confirmed by the MTT-test. To study airway provocations cumulative concentration-response curves were performed with different allergic response mediators and biogenic amines. In addition, electric field stimulation, passive sensitization with house dust mite (HDM) and mast cells staining were evaluated.

**Results:**

PCLS from sheep were viable for at least three days. PCLS of newborn and adult sheep responded equally strong to methacholine and endothelin-1. The responses to serotonin, leukotriene D_4_ and U46619 differed with age. No airway contraction was evoked by histamine, except after cimetidine pretreatment. In response to EFS, airways in PCLS from adult and newborn sheep strongly contracted and these contractions were atropine sensitive. Passive sensitization with HDM evoked a weak early allergic response in PCLS from adult and newborn sheep, which notably was prolonged in airways from adult sheep. Only few mast cells were found in the lungs of non-sensitized sheep at both ages.

**Conclusion:**

PCLS from sheep lungs represent a useful tool to study pharmacological airway responses for at least three days. Sheep seem well suited to study mechanisms of cholinergic airway contraction. The notable differences between newborn and adult sheep demonstrate the importance of age in such studies.

## Introduction

The prevalence of asthma increased during the last decades [1], [2] and may be related to Western lifestyle factors [2], [3]. However, the causal reasons and underlying mechanisms are not well understood. Several studies have shown that in most cases of persistent asthma, the initial asthma-like symptoms occur during the first years of life [4], [5]. It has been suggested that the child's environment plays an important role to develop asthma later in life. Children exposed to a farm environment had less asthma and atopy than children grown-up in a non-farming setting [6]–[9]. The mechanism associated with this protective effect is unknown. It is suggested that the child's immune system may be stimulated along a Th1 pathway by early exposure to increased concentrations of bacterial components present in stables such as endotoxin (LPS) [10]. This theory is known as the hygiene hypothesis [11]. For some of these children asthma symptoms seem to remit with time, but many children develop asthmatic symptoms which persist throughout their life and are associated with more severe symptoms ending in the loss of lung function. About 15% of the wheezing infants develop persistent wheezing and clinical asthma later in life [12].

Animal models of asthma should display the pathology of the human disease and have carefully to be selected. Several studies indicate for instance that the innervation of the lung differs considerably between and within species [13], [14]. Moreover, rodent airways do not or only weakly respond to leukotrienes [15], mediators that readily cause bronchoconstriction in humans [16] and guinea pigs [17]. Sheep show a greater resemblance to humans concerning lung development compared to rodents. Rodents and guinea pigs undergo the alveolar phase of lung development postnatally whereas sheep and humans undergo this phase in the uterus [18]–[20]. In addition sheep can, like rodents, be sensitized to house dust mite (HDM) antigen which is a common human antigen in asthma, and have allergen-specific IgE responses and acute eosinophil responses to allergen challenge [21], [22]. Therefore, sheep have been considered particularly useful as models to study allergic airway responses to common human natural antigens.

Airway responses can be visualized by precision-cut lung slices (PCLS), which are viable lung tissue slices of uniform thickness (≈250 µm). PCLS can easily be prepared from different species and are already established for many species including rat, mouse, guinea pig, non-human primates and humans [14], [17], [23]–[26]. PCLS represent a highly useful model to study bronchial and pulmonary vascular responses by videomicroscopy [23], [27]. The responses in pulmonary vessels strips of newborn and adult sheep have shown interesting differences in reactivity [28]. The diameter of the pulmonary vessels increased from the newborn to the adult animals and the maximum velocity of shortening was in newborns much higher than in adult sheep. The responses of airways in newborn and adult sheep have not yet been investigated. We hypothesize that differences in airway smooth muscle reactivity in sheep are present at different ages.

In the present study the bronchoconstriction of newborn and adult sheep was studied in PCLS. A rationale of this study was to establish a model of ovine PCLS for the measurement of airway responses to early allergic response mediators and to allergens (after passive sensitization) in newborn and in adult animals.

## Materials and Methods

### Animal model

All animal procedures were approved by the Animal Ethics Committee of the University of Maastricht, The Netherlands. The newborns (n = 7) were delivered spontaneously at term (147±2 days GA). The lambs were euthanized directly after surgical delivery with an intra−venous injection of pentobarbital. Adult sheep (n = 5) were euthanized at ∼18 months with pentobarbital.

### Preparation of PCLS

PCLS were prepared from adult or newborn sheep lung as described in previous studies [14], [17], [23]–[25] with some modifications. Briefly, the lungs were filled via the lobular bronchus with 1.5% low-melting-point agarose solution and put onto ice until the agarose had solidified. Tissue cores (1 cm in diameter) with a penetrating airway were punched out. Those cylinders were then cut perpendicular to the airway by means of a Krumdieck tissue slicer (Alabama Research and Development, Munford, AL, USA) into approximately 250 µm thin PCLS. PCLS were transferred into a 10 cm cell culture dish and incubated under cell culture conditions (37°C, 5% CO_2_ atmosphere) in minimal essential medium (MEM) that was frequently changed during the next 4 hours (h) and incubated over night. The medium exchange supported the removal of tissue released mediators as well as the wash out of agarose from airways. For measurements, only slices with airways free of agarose, with beating cilia and an intact and relaxed airway smooth muscle layer were used. We only studied PCLS with comparable airway size in parallel experiments to reduce interslice variations. Electric field stimulation (EFS) on PCLS and passive sensitization studies were performed within 24 h after preparation. All other physiological measurements were conducted within 48 h after preparation.

### Viability of PCLS

Viability of PCLS over three day incubation was confirmed by intracellular reduction of a tetrazolium dye to its according purple formazan (MTT-test) and constriction responses. PCLS were transferred into cavities of a standard 24−well plate (1 PCLS/well) and incubated with 900 µL MEM + 100 µL 3−(4,5−dimethylthiazol−2−yl)−2,5−diphenyl tetrazolium bromide solution (MTT, 7 mg/mL) for 15 min. The supernatant was discarded and the formazan was dissolved by incubation of PCLS with 200 µL formic acid/propanol (5%/95%) solution for 20 min. 100 µL of the purple supernatant were taken and transferred to 96−well plates to measure the extinction at 550 nm (Tecan GENios Microplate Reader). All reactions were carried out at room temperature and in the dark. For negative control measurements PCLS were digested with 0.2% (v/v) Triton−X100 (300 µL, 20 min, 37°C) before performing the MTT−assay.

### Videomicroscopy

If not otherwise stated, PCLS were kept in cavities of standard 24-well plates and were immersed in 1 mL MEM during the experiment. The plate was then mounted on the stage of an inverted Leica DMIL microscope (Leica Microsystems, Wetzlar, Germany). The airways were imaged and digitized by videomicroscopy (SensiCam 365KL digital camera, Visitron Systems, Munich, Germany; Optimas 6.5 software, Optimas, Bothell, WA, USA). The airway area before any provocation was defined as 100%−initial airway area [%−IAA].

### Airway provocations by early allergic response mediators and biogenic amines

Cumulative concentration-response curves were performed with methacholine (10^−10^ M–10^−4^ M; 5 min/conc.; pictures every 5 s), serotonin (10^−10^ M–10^−4^ M; 5 min/conc.; pictures every 5 s), histamine (10^−9^ M–10^−4^ M; 5 min/conc.; pictures every 5 s), endothelin-1 (10^−12^ M–10^−6^ M; 10 min/conc.; pictures every 10 s), leukotriene D4 (LTD_4_, 10^−12^ M–10^−6^ M; 10 min/conc.; pictures each 5 s) and the thromboxane A_2_ analogue U46619 (10^−10^ M–10^−5^ M; 10 min/conc.; pictures every 5 s). To study a potential effect of the histamine H_2_-receptor on bronchoconstriction, PCLS were pre-incubated with 10 µM cimetidine for 15 min prior to performing the histamine concentration-response curve.

### Electric field stimulation (EFS)

As described before [29], EFS of PCLS was carried out in standard 12-well plates at a reaction volume of 1 mL standard MEM. The PCLS were placed in between two platinum electrodes of 12 mm distance and were mounted by a Teflon ring. The electric field was applied by a Hugo Sachs Electronics Stimulator II (Hugo Sachs Electronics, March Hugstetten, Germany). The electric stimuli were defined by a frequency of 50 Hz, pulse duration of 1 ms, a current amplitude of 200 mA, a train width of 2.5 s and a train rhythm of 60 s. Each train lasted 3.3 min. After the first control stimulation the muscarinic antagonist atropine (10 µM) was added and incubated for 15 min prior to the second stimulation. In an additional set of EFS experiments, frequency response curves were conducted on PCLS from adult and newborn sheep. In this, the frequency was steadily increased from 0.4 Hz–100 Hz, while the pulse duration and current amplitude were kept constant at 1 ms and 200 mA, respectively. Each frequency was applied once for 2.5 s and after a pause of one minute the next frequency was applied. The airway behavior of PCLS upon stimulation was monitored by videomicroscopy. Pictures were taken every 2.5 s. Airway area before the first stimulation was defined as 100% initial airway area (IAA).

### Passive sensitization

Passive sensitization was performed by incubation with 1% serum from house dust mite (HDM)−sensitized sheep overnight. Medium was replaced by fresh serum−free MEM directly before provocation with 5000 Units of HDM from ALK−SHERAX (Wedel, Germany), which is normally used for intracutaneous testing of atopy. Airway responses were followed by videomicroscopy for 20 min. Pictures were taken every 10 s and pruned for clarity to each fifth data point.

### Mast cell staining

Mast cells were stained with toluidine blue or with alcian blue and safranin. Briefly, PCLS were fixed with formaldehyde 4% (Roti−Histofix 4%, Carl Roth GMBH & Co. KG, Karlsruhe, Germany), dehydrated and embedded in paraffin. Then, 5 µm thick sections were cut, deparaffinized, rehydrated and incubated in 1% toluidin blue for 30 min. After rinsing with water, sections were dehydrated and coverslipped. Alternatively after rehydration, sections were incubated in 0.05% Alcian Blue in 0.02 M acetate buffer, pH 5.8 with 0.2 M MgCl for 4 h. After rinsing in water, sections were incubated for 5 min in 0.25% safranin 0.02 M acetate buffer, pH 5.0. After a final rinsing step, sections were dehydrated and coverslipped.

### Data analysis

Data are shown as means ± standard error of the mean (SEM). Concentration− response curves were fitted by non-linear regression (4−parameter logistic equation). To analyze differences between curves, each parameter of the 4−parameter logistic equation was separately compared by the Extra sum-of-squares F-test. The 4-parameter logistic equation is [Y = Bottom + (Top-Bottom)/(1+10∧((LogEC50-X)*HillSlope)], with the bottom being the maximal response (i.e. fit values aim towards the minimum in the concentration response curves), the top being the initial situation (i.e. fit values aim towards 100%-IAA), the logEC50 being calculated to the half maximal response and the slope initially kept variable (i.e. unconstrained from a standard value of 1). A shared concentration-response curve was plotted for adult and newborn sheep, if no difference was found in any parameter of the 4−parameter logistic equation. Minimal airway areas in EFS before and after atropine treatment were compared by Student‘s t−test (due to homogeneity of variance). Mixed model analysis considering changes in airway area dependent on time and treatment was performed on time courses in the passive sensitization experiments. P-values <0.05 were considered significant. The statistical analysis was performed by either GraphPad Prism 5 (GraphPad Software, La Jolla, CA) or SAS 9.1 (SAS Institute Inc., Cary, NC).

## Results

### Viability of PCLS

**Figure 1:**
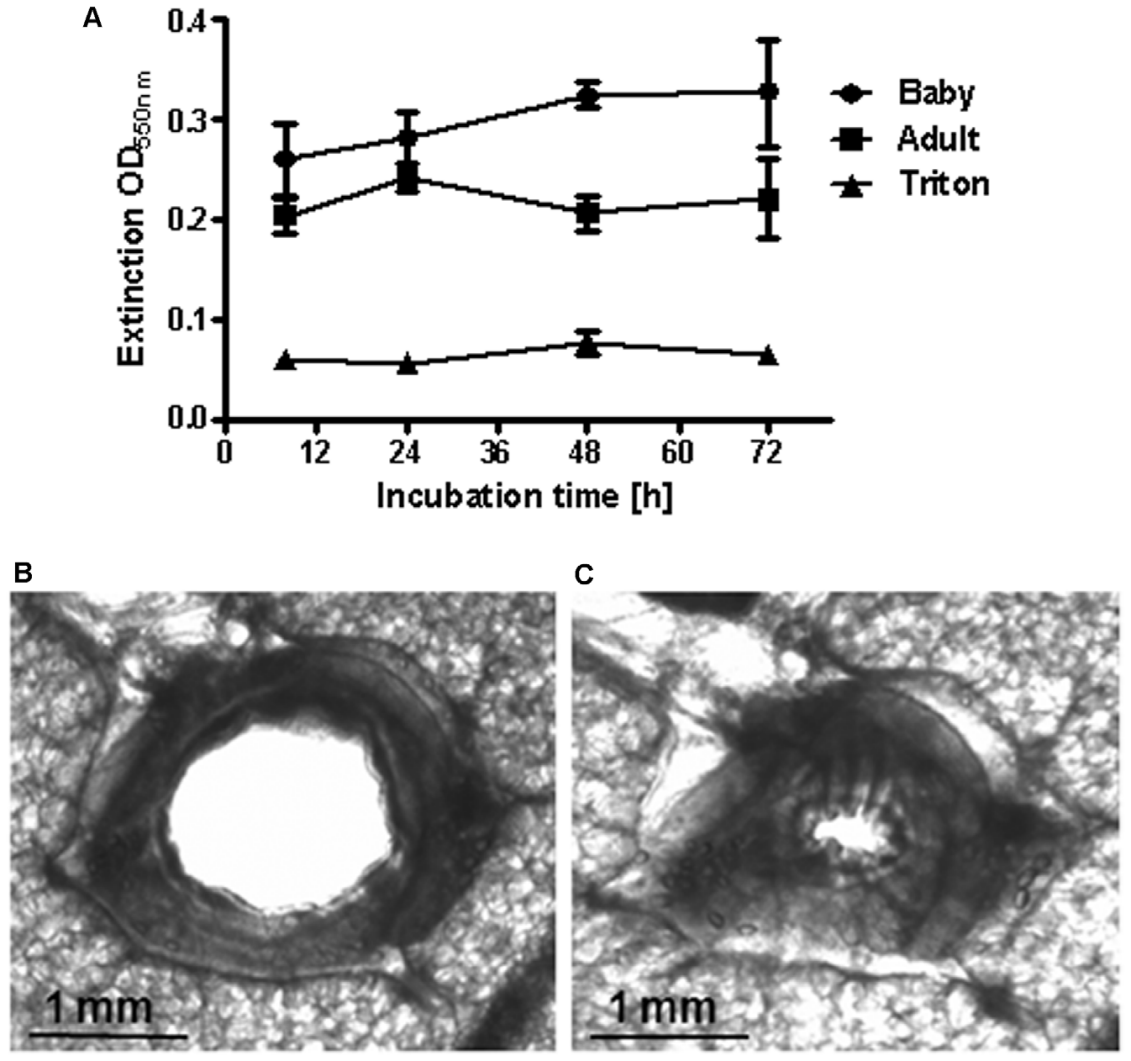
Viability of sheep PCLS. **A**) Viability was followed by the MTT test. High extinctions indicate high viability. Data are shown as mean ± SEM, n = 3 PCLS from 3 animals per group. Exemplary photographs (in black and white) of a PCLS before (**B**) and after (**C**) provocation with 10^−4^ M metacholine.

More than 50 slices were obtained from one lung lobe of either newborn or adult sheep. The viability of sheep PCLS was demonstrated by the MTT test and PCLS were viable for at least three days (figure 1). In figure 1A, high extinctions in this test indicate intact cellular reduction systems, which correlate to viability. PCLS of adult sheep had an extinction OD of 0.2 stable over 3 days compared to deterged control PCLS with an extinction OD of 0.05. PCLS of newborn sheep had also a robust extinction OD of up to 0.3 over 3 days. Functionality of airway smooth muscle contraction was shown by the application of 10^−4^ M methacholine, which evoked a strong bronchoconstriction in sheep PCLS (figure 1B and C).

### Airway provocations by allergic response mediators or biogenic amines in adult and newborn sheep

**Figure 2:**
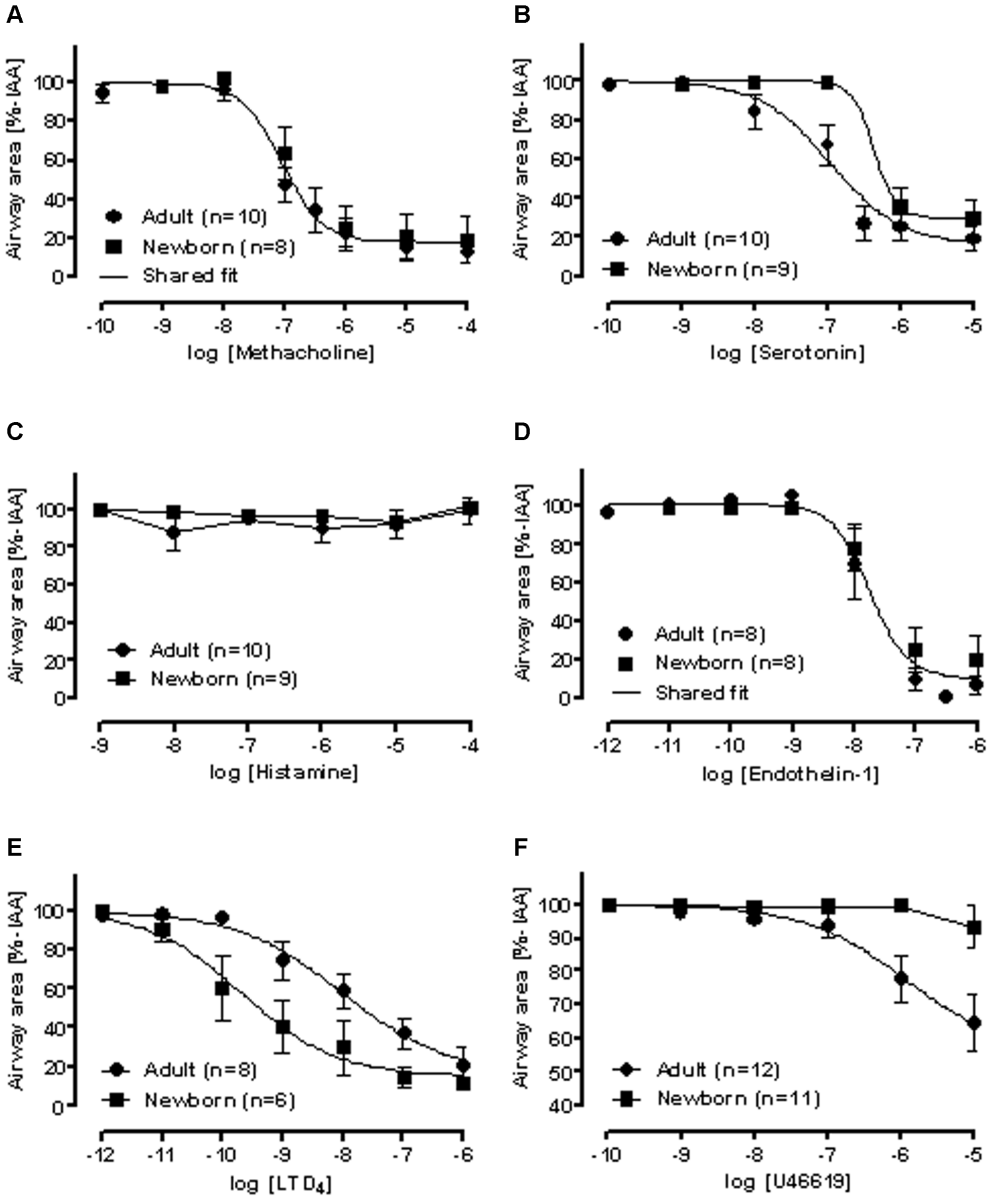
Concentration-response curves for common mediators of early allergic response in PCLS of adult (•) and newborn sheep (▪). **A**) methacholine, **B**) serotonin, **C**) histamine, **D**) endothelin-1, **E**) leukotriene D_4_ (LTD_4_), and **F**) U46619. n  =  number of PCLS, whereby a total of 7 newborn and 5 adult sheep were examined. Data are shown as mean ± SEM. A shared concentration-response curve was plotted for adult and newborn sheep, if no difference was found in any parameter of the 4−parameter logistic equation.

In sheep PCLS all of the mediators of the early allergic response in humans, except for histamine, evoked a marked bronchoconstriction (figure 2A−F). In general, concentration-response curves are characterized by their efficacy and potency. Shared concentration-response curves in PCLS from adult and newborn sheep were found for methacholine and endothelin-1 with half-maximal responses (log EC_50_ [M]) of −7.0±0.1 and −7.7±0.1, respectively (figure 2A and 2D). Age related differences in potency were found for serotonin and LTD_4_. For serotonin the logEC_50_ [M] values were −6.3±0.4 for newborn and −6.9±0.2 for adult sheep (figure 2B), whereas for LTD_4_ logEC_50_ values were smaller for newborn than for adult sheep (−9.7±0.4 [M] vs. −8.0±0.6 [M]) (figure 2E). Differences in efficacy were found for U46619, as airways of adult sheep responded markedly to U46619 (logEC_50_ [M]  = −5.9±1.0; max contraction to 64.6±8.4%−IAA at 10^−5^ M), whereas airways of newborn sheep were mostly unresponsive (max. contraction to 93.4±6.5%−IAA at 10^−5^ M) (figure 2F).

### Airway provocation by histamine with or without cimetidine

**Figure 3:**
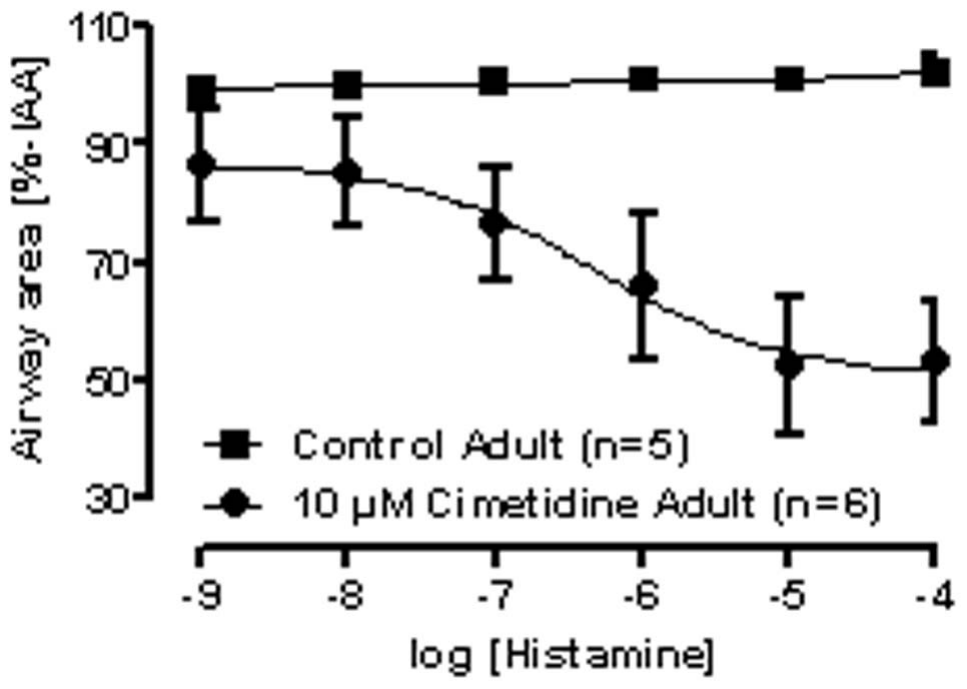
H_2_−receptor counteracts histamine-induced bronchoconstriction in adult sheep PCLS. PCLS from adult sheep were either pre-incubated with the H_2_-receptor antagonist cimetidine before histamine provocation (•) or kept untreated (control, ▪). Data are shown as mean ± SEM, n  =  number of PCLS of separate sheep. The concentration response curves were fitted by the 4−parameter logistic equation.

Since in the preceding experiments no airway contraction was observed by histamine, PCLS were pretreated with or without cimetidine, which blocks the H_2_−receptor. Again, airways in control PCLS, i.e. without cimetidine, did not contract in adult sheep (figure 3). In contrast, stimulation of airways in PCLS with histamine after cimetidine pretreatment resulted in a marked concentration-dependent contraction (logEC_50_ = −6.3) (figure 3). No data were available for PCLS from newborn sheep.

### Intrinsic activation of bronchoconstriction in sheep PCLS

In addition to the preceding exogenous activation of bronchoconstriction in PCLS by exogenous application of mediators, we studied whether an intrinsic activation of airway response is possible in sheep PCLS. In this regard, two approaches were chosen: EFS to study neutrally-induced bronchoconstriction and passive sensitization to evoke an early allergic response by mast cell degranulation after antigen challenge.

**Figure 4:**
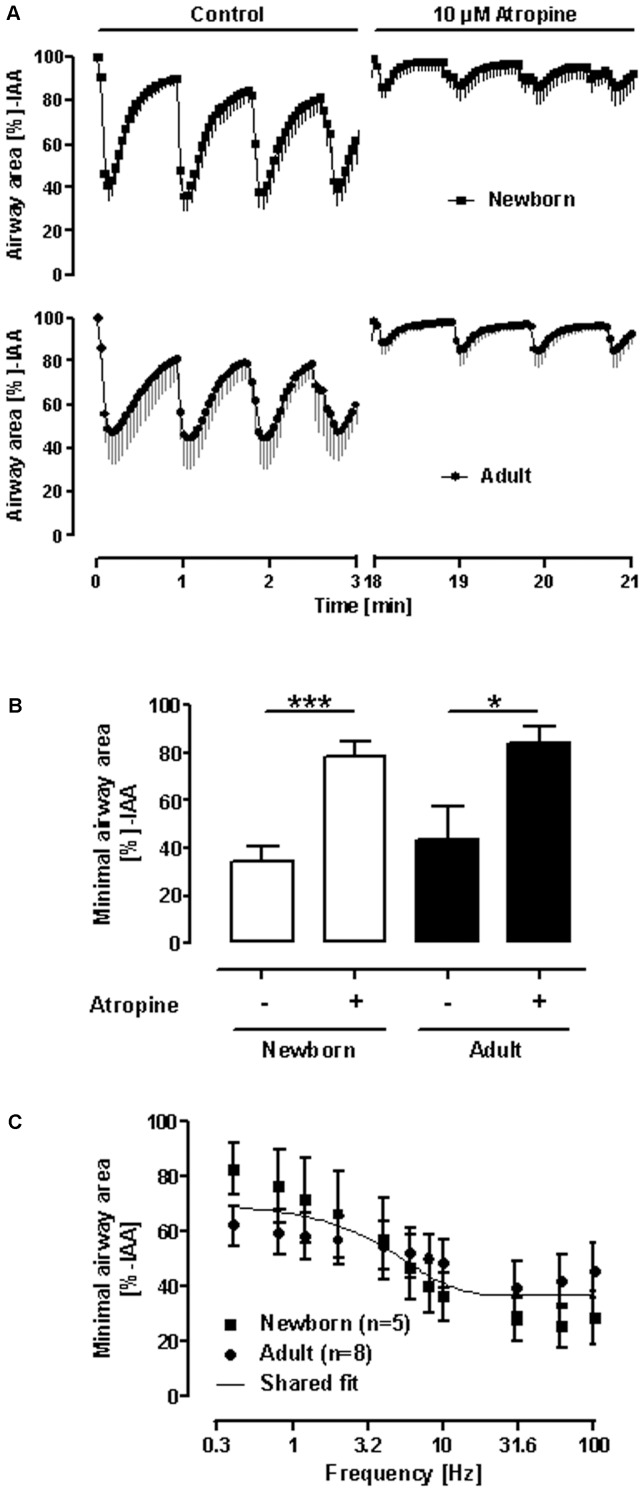
Electric field stimulation (EFS) of PCLS from newborn and adult sheep. **A**) Course of airway area changes during repeated EFS in absence or presence of atropine. **B**) Statistical analysis on minimal airway area during EFS as obtained in **A**. Data are shown as mean ± SEM, n = 7 for newborn sheep and n = 4 for adult sheep, whereby each PCLS was taken from an independent sheep; * p<0.05; *** p<0.001 in Student‘s t−test. **C**) Frequency-response curves of EFS-induced airway contractions. Data are shown as mean ± SEM, n = 5 PCLS from three newborn sheep and n = 8 PCLS from eight adult sheep. A shared frequency-response curve was plotted for adult and newborn sheep, since there was no difference in any parameter of the 4−parameter logistic equation assuming that the top is equal to 100%-IAA and EF_50_ is larger than zero Hz.

In response to EFS, PCLS from both adult and newborn sheep, strongly contracted to 43.7±13.9% IAA and 34.3±6.5% IAA (figure 4), respectively. This neurally−induced response was potently blocked by atropine resulting in an airway contraction to only 84.0±7.6% IAA for PCLS from adult sheep and 78.6±6.8 for PCLS from newborn sheep.. Moreover, to distinguish between a difference in sensitivity of PCLS from newborn and adult sheep to EFS, frequency response curves were conducted. However, a shared curve was found (figure 4 C), indicating equal sensitivity of PCLS from newborn and adult sheep to neural stimulation.

**Figure 5:**
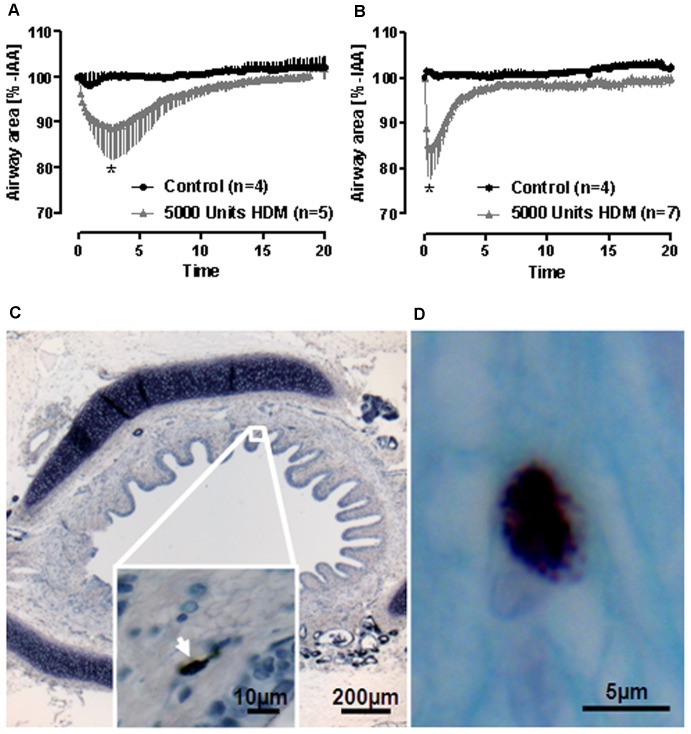
Early allergic response in sheep PCLS after passive sensitization. PCLS from newborn (**A**) or adult (**B**) sheep were passively sensitized with serum from actively sensitized sheep against HDM and provoked with 5000 U HDM. Data are shown as mean ± SEM, n as indicated on PCLS from different sheep. Time courses were statistically compared by mixed model analysis; *p<0.05. If one compares HDM groups in A and B, courses are also significant different, which points out differences in the kinetics of allergen-induced bronchoconstriction in adult and newborn sheep. **C, D**) Less than one mast cell per 5 µm thick section were found in PCLS from sheep. PCLS were stained for mast cells with toluidine blue (**C, arrow**) or with alcian blue and safranin (**D**).

With respect to passive sensitization, provocation with 5000 U of HDM evoked a weak but significant early allergic response in PCLS from adult and newborn sheep, that had been incubated with serum from HDM sensitized sheep (figure 5A and 5B). Comparing the HDM-induced airway contractions in PCLS from adult and newborn sheep, a significant difference was found in the respective kinetics. The response to HDM was more sustained in adult than in newborn sheep. In order to study these responses, we stained for mast cells in the lung tissue since we consider them essential for the response. In accordance to the weak response, very few mast cells were found in PCLS from sheep at both ages as shown in figure 5C and 5D. We found on average less than one mast cell per 5 µm thick section.

## Discussion

To our knowledge this is the first time, the airway reactivity of newborn and adult sheep was compared in PCLS. Many of the responses were similar in newborn and adult sheep, such as the bronchoconstriction to EFS, and to the mediators methacholine, histamine and endothelin-1. On the other hand different responses in newborn and adult sheep were found for the eicosanoids LTD_4_ and thromboxane as well as the biogenic amine serotonin. The response to allergen after passive sensitization was comparable in the extent of contraction, but prolonged in adult sheep.

PCLS are viable, can readily be obtained from different species and offer a great opportunity to study airway responses under cell culture conditions [14]. The microarchitecture of lung tissue, i.e. airways and vessels embedded in the parenchyma, remains intact and permit physiological measurements [23]–[26]. Even nerves remain functional and can be specifically activated [29]. In the past, PCLS from rat, mouse, guinea pig, non-human primate and human lung have been investigated [14], [26]. Allergic responses in sheep have been suggested to be similar to humans [18], [19], [21], [22], but the response of airways to early allergic response mediators in newborn compared to adult sheep had not been investigated yet. Recurrent wheezing is common in young infants and toddlers [30]. For some of these children asthma symptoms seem to remit with time, but many children develop asthmatic symptoms which persist throughout their life [12], [31]. Therefore, it is important to compare the allergic responses in newborn to adult lung tissue.

Given the importance of small airways for asthma and COPD [24], [25], [32], [33], the possibility to study these airways in PCLS is of clinical relevance. Small airways are defined by their inner diameter being less than 2 mm in human (airway generations >11) and less than 780 µm in rat airways [25], [34]. Since sheep are comparable in weight to humans, lung/airway sizes and generations should correlate to the human structure. In this context of note, the current experiments were exclusively performed on small airways, since they were less than 2 mm in diameter. Therefore, our results reflect the situation only for the peripheral lung reducing interslice variations since airway responses can differ between large and small airways [23], [29].

In the present study PCLS from sheep were viable for at least three days, not different from the viability of human PCLS [24], making it possible to study the effects of cytokines, growth factors and maybe even remodeling processes [35], [36]. In addition, recent work by Behrsing and colleagues [37] has demonstrated that the process of slicing itself elicits a massive cytokine response, confounding early results of cytokine measurements in response to PCLS challenge. Moreover, in contrast to our study, they also used transcriptionally active agents like humulin, hydrocortisone and retinoic acid in their incubation medium. In the current study, we have performed frequent medium exchanges during the first 24 h after slicing to minimize the effect of endogenously released mediators. Nonetheless in general, the impact of mechanical damage conducted to create the PCLS should be noticed when cytokines and mediators are studied in PCLS.

PCLS of newborn and adult sheep responded very similar to methacholine and endothelin-1, mediators also effective in humans [17]. However, the response to serotonin, the thromboxane analogue U46619 and LTD_4_ depended on the age of the sheep. In newborn sheep the response to serotonin and U46619 was weaker than in adult sheep, whereas the response to LTD_4_ was stronger in newborn sheep. This is an important difference compared to rodent airways that do not or at best very weakly respond to LTD_4_ [15]. Leukotrienes contribute to airway obstruction in many human asthmatics [29]. In this regard sheep PCLS, in particular newborn, may appear as a suitable animal model to study bronchoconstriction. From pharmacological and clinical point of view these results are very interesting. The strong bronchial response of newborn sheep to leukotrienes suggests that young animals will be more sensitive to the effects of montelukast, which is a leukotriene-receptor antagonist and used to prevent the wheezing and shortness of breath caused by asthma. On the other hand, in the present study it is demonstrated that adult sheep have a stronger bronchial response to serotonin. Therefore, adult sheep may be more sensitive to the effects of ketanserin, which is a highly selective antagonist for contractile serotonin 5-HT_2A_ receptors. Our studies show a lifetime dependent response pattern to different antagonist, which suggest specific disease treatment with different antagonist depending on the age of the sheep.

Compared to human PCLS, there are also differences: sheep airways reacted to serotonin rather than to histamine, which is the opposite to what is observed in human and guinea pigs PCLS, in which histamine (EC50 2.7 mM) is effective but serotonin is not [17], [24], [25]. The reason for these species differences is not clear. It was suggested that it may, at least in part, be related to age differences in tissue as shown in young healthy guinea pigs versus middle-aged adults [17], [24], [25]. However, the present study demonstrated that the bronchoconstriction in both newborn and adult sheep was the same and that age differences may not play such a crucial role if one compares responses from animal tissue with that from human tissue.

Importantly, however, the lack of responsiveness to histamine was not due to the lack of H_1_-receptors. This was revealed by experiments with the H_2_-receptor antagonist cimetidine (figure 3), which has also minor affinity for H_1_-, H_3_- and H_4_-receptors [38], [39]. Here, we consider effects of H_3_ and H_4_ activities unlikely, because H_3_ is predominantly expressed in brain tissue and H_4_ is attributed to immune cell responses [40], [41]. Moreover, in literature there are no reports about H_3_ and H_4_ expression in sheep lung. Since no analysis of the different histamine receptor expression was performed in lung tissue in the present study the discussion about H_3_ and H_4_ remains speculative and is a clear limitation of this study. Normally, in the lungs histamine acts on H_1_- and H_2_-receptors and its effect is therefore balanced by the H_1_-mediated contraction and the H_2_-mediated relaxation of airway smooth muscles [42]–[45]. The observation that histamine contracts tracheal and bronchial airways, but relaxes smaller bronchi and bronchioles [46], indicates that there is a shift towards predominance of H_2_−receptors towards the peripheral airways. Thus, the present finding that histamine was able to contract ovine airways only after blockade of H_2_-receptors, may reflect the fact that the PCLS were derived from the lung periphery. In addition, the expression of histamine H_2_-receptors in the lungs may also differ depending on the type of lung sample, previous sensitization or infection, frequently leading to down-regulation of H_2_-receptors [47]–[49]. Therefore, H_2_-receptor deficiency in the airways may, at least in part, explain the commonly observed airway hyperreactivity to histamine in asthmatics.

In the present study, the early allergic response in passively sensitized sheep PCLS was weak. One explanation might be that after degranulation mast cells mainly release histamine, which – as seen before – is ineffective unless H_2_-receptors are blocked. Alternatively, this finding can be explained by the small number of mast cells found in the airways of PCLS from non-sensitized sheep. It was demonstrated that exposure to HDM (active sensitization) doubled the number of mast cells mostly in alveolar septa and in airway walls [22], [50]. Notably, these responses required at least 16 weekly HDM challenges [50], while in the present study the sheep lungs were unchallenged and PCLS were only passively sensitized with serum from actively sensitized sheep. This might explain the small numbers of mast cells in the present study. Another interesting observation in the allergen-induced bronchoconstriction after passive sensitization was the prolonged airway contraction in PCLS from adult sheep compared to newborn sheep. To our knowledge this is the first study examining the early allergic response of adult and newborn animals in parallel. Reasons, such as a sustained mediator release, diminished degradation of bronchoconstrictors or different receptor density in adult sheep, are therefore speculative. Nonetheless, this example confirms, that extreme caution is mandated selecting the appropriate animal model, when studying childhood asthma or asthma in adults as responses may differ tremendously.

We further demonstrated that ovine PCLS respond to electric field stimulation. Airway responses to electric field stimulation differ largely between species [14]. PCLS from rats and marmoset airways contract by about 20%, whereas guinea pigs, sheep and humans contract at maximum by about 40–60% [14]. The present study adds that the maximum contraction (>50%) in both newborn and adult sheep did not differ. Moreover, since the sensitivity of sheep PCLS from newborn and adult sheep did not differ, one may speculate that the neural network in sheep lung is widely established by birth. The shared frequency-response curve is also in line with the exogenous application of methacholine, in which also a shared curve, i.e. same sensitivity to the agonist, was found. These findings are rounded off by the high atropine-sensitivity of EFS-induced airway contractions pointing out that lung innervation in sheep is mostly cholinergic. Hence, airway responses in sheep PCLS are a reasonable proxy for human airways, if cholinergic airway contractions are intended to study.

In our model we focused on the acute response to exogenously added inflammatory mediators and passive HDM sensitization to demonstrate that ovine PCLS are a reliable tool for in vitro measurement of airway responses. It was beyond the scope of this study to examine the role of cytokine and mediator secretion in response to a physiologically relevant stimulus or active sensitization. In future research it will be relevant to compare acute versus chronic responses after HDM sensitization and also to compare HDM to sensitization with other allergens. In conclusion, PCLS from sheep lungs represent a useful tool to study pharmacological airway responses for at least three days. Sheep seem well suited to study mechanisms of cholinergic airway contraction. Their airway pharmacology differs in some respects to that observed in humans. Bronchoconstriction is similar in newborn and adult sheep, except for the lipid mediators (LTD4, U46619) and serotonin. Early allergic response is weak which is probably based on a small number of mast cells.

## References

[1] Manning PJ, Goodman P, O'Sullivan A, Clancy L (2007) Rising prevalence of asthma but declining wheeze in teenagers (1995–2003): ISAAC protocol. Ir Med J 100: 614–615. 18277728

[2] Beasley R, Crane J, Lai CK, Pearce N (2000) Prevalence and etiology of asthma. J Allergy Clin Immunol 105: S466–472. 10669525 10.1016/s0091-6749(00)90044-7

[3] Britton J (2003) Parasites, allergy, and asthma. Am J Respir Crit Care Med 168: 266–267. 12888603 10.1164/rccm.2305011

[4] Martinez FD, Wright AL, Taussig LM, Holberg CJ, Halonen M, et al (1995) Asthma and wheezing in the first six years of life. The Group Health Medical Associates. N Engl J Med 332: 133–138. 7800004 10.1056/NEJM199501193320301

[5] Barbee RA, Dodge R, Lebowitz ML, Burrows B (1985) The epidemiology of asthma. Chest 87: 21S–25S. 3871083 10.1378/chest.87.1.21s

[6] Von Ehrenstein OS, Von Mutius E, Illi S, Baumann L, Bohm O, et al (2000) Reduced risk of hay fever and asthma among children of farmers. Clin Exp Allergy 30: 187–193. 10651770 10.1046/j.1365-2222.2000.00801.x

[7] Riedler J, Eder W, Oberfeld G, Schreuer M (2000) Austrian children living on a farm have less hay fever, asthma and allergic sensitization. Clin Exp Allergy 30: 194–200. 10651771 10.1046/j.1365-2222.2000.00799.x

[8] Braun-Fahrlander C, Gassner M, Grize L, Neu U, Sennhauser FH, et al (1999) Prevalence of hay fever and allergic sensitization in farmer's children and their peers living in the same rural community. SCARPOL team. Swiss Study on Childhood Allergy and Respiratory Symptoms with Respect to Air Pollution. Clin Exp Allergy 29: 28–34. 10051699 10.1046/j.1365-2222.1999.00479.x

[9] Riedler J, Braun-Fahrlander C, Eder W, Schreuer M, Waser M, et al (2001) Exposure to farming in early life and development of asthma and allergy: a cross-sectional survey. Lancet 358: 1129–1133. 11597666 10.1016/S0140-6736(01)06252-3

[10] von Mutius E, Braun-Fahrlander C, Schierl R, Riedler J, Ehlermann S, et al (2000) Exposure to endotoxin or other bacterial components might protect against the development of atopy. Clin Exp Allergy 30: 1230–1234. 10971468 10.1046/j.1365-2222.2000.00959.x

[11] Renz H, Blumer N, Virna S, Sel S, Garn H (2006) The immunological basis of the hygiene hypothesis. Chem Immunol Allergy 91: 30–48. 16354947 10.1159/000090228

[12] Guilbert T, Krawiec M (2003) Natural history of asthma. Pediatr Clin North Am 50: 523–538. 12877234 10.1016/s0031-3955(03)00044-0

[13] Abraham WM (2000) Animal models of asthma. In: Busse WW, Holgate ST, editors. Asthma and rhinitis. Oxford; Malden, MA, USA: Blackwell Science. pp. 1205–1227.

[14] Schleputz M, Rieg AD, Seehase S, Spillner J, Perez-Bouza A, et al (2012) Neurally Mediated Airway Constriction in Human and Other Species: A Comparative Study Using Precision-Cut Lung Slices (PCLS). PloS one 7: e47344. 23056631 10.1371/journal.pone.0047344 PMC3467211

[15] Held HD, Martin C, Uhlig S (1999) Characterization of airway and vascular responses in murine lungs. Br J Pharmacol 126: 1191–1199. 10205008 10.1038/sj.bjp.0702394 PMC1565872

[16] Dahlen SE, Hedqvist P, Hammarstrom S, Samuelsson B (1980) Leukotrienes are potent constrictors of human bronchi. Nature 288: 484–486. 6108512 10.1038/288484a0

[17] Ressmeyer AR, Larsson AK, Vollmer E, Dahlen SE, Uhlig S, et al (2006) Characterisation of guinea pig precision-cut lung slices: comparison with human tissues. Eur Respir J 28: 603–611. 16737991 10.1183/09031936.06.00004206

[18] Kramer BW (2011) Chorioamnionitis - new ideas from experimental models. Neonatology 99: 320–325. 21701204 10.1159/000326620

[19] Pringle KC (1986) Human fetal lung development and related animal models. Clin Obstet Gynecol 29: 502–513. 3757332

[20] Wolfs TG, Jellema RK, Turrisi G, Becucci E, Buonocore G, et al (2012) Inflammation-induced immune suppression of the fetus: a potential link between chorioamnionitis and postnatal early onset sepsis. J Matern Fetal Neonatal Med 25 Suppl 18–11. 22348330 10.3109/14767058.2012.664447

[21] Bischof RJ, Snibson K, Shaw R, Meeusen EN (2003) Induction of allergic inflammation in the lungs of sensitized sheep after local challenge with house dust mite. Clin Exp Allergy 33: 367–375. 12614452 10.1046/j.1365-2222.2003.01534.x

[22] Snibson KJ, Bischof RJ, Slocombe RF, Meeusen EN (2005) Airway remodelling and inflammation in sheep lungs after chronic airway challenge with house dust mite. Clin Exp Allergy 35: 146–152. 15725184 10.1111/j.1365-2222.2005.02137.x

[23] Martin C, Uhlig S, Ullrich V (1996) Videomicroscopy of methacholine-induced contraction of individual airways in precision-cut lung slices. Eur Respir J 9: 2479–2487. 8980957 10.1183/09031936.96.09122479

[24] Wohlsen A, Martin C, Vollmer E, Branscheid D, Magnussen H, et al (2003) The early allergic response in small airways of human precision-cut lung slices. Eur Respir J 21: 1024–1032. 12797499 10.1183/09031936.03.00027502

[25] Wohlsen A, Uhlig S, Martin C (2001) Immediate allergic response in small airways. Am J Respir Crit Care Med 163: 1462–1469. 11371419 10.1164/ajrccm.163.6.2007138

[26] Seehase S, Schleputz M, Switalla S, Matz-Rensing K, Kaup FJ, et al (2011) Bronchoconstriction in nonhuman primates: a species comparison. J Appl Physiol 111: 791–798. 21700889 10.1152/japplphysiol.00162.2011

[27] Rieg AD, Rossaint R, Uhlig S, Martin C (2011) Cardiovascular agents affect the tone of pulmonary arteries and veins in precision-cut lung slices. PloS one 6: e29698. 22216346 10.1371/journal.pone.0029698 PMC3246495

[28] Belik J, Halayko A, Rao K, Stephens N (1991) Pulmonary vascular smooth muscle: biochemical and mechanical developmental changes. J Appl Physiol 71: 1129–1135. 1757309 10.1152/jappl.1991.71.3.1129

[29] Schleputz M, Uhlig S, Martin C (2011) Electric field stimulation of precision-cut lung slices. J Appl Physiol (1985) 110: 545–554. 21109600 10.1152/japplphysiol.00409.2010

[30] Robison RG, Singh AM (2012) Chapter 11: the infant and toddler with wheezing. Allergy Asthma Proc 33 Suppl 1S36–38. 10.2500/aap.2012.33.3543 22794684

[31] Been JV, Lugtenberg MJ, Smets E, van Schayck CP, Kramer BW, et al (2014) Preterm birth and childhood wheezing disorders: a systematic review and meta-analysis. PLoS Med 11: e1001596. 24492409 10.1371/journal.pmed.1001596 PMC3904844

[32] Sturton RG, Trifilieff A, Nicholson AG, Barnes PJ (2008) Pharmacological characterization of indacaterol, a novel once daily inhaled 2 adrenoceptor agonist, on small airways in human and rat precision-cut lung slices. J Pharmacol Exp Ther 324: 270–275. 17916760 10.1124/jpet.107.129296

[33] Sturton G, Persson C, Barnes PJ (2008) Small airways: an important but neglected target in the treatment of obstructive airway diseases. Trends Pharmacol Sci 29: 340–345. 18514920 10.1016/j.tips.2008.04.003

[34] Yeh HC, Schum GM, Duggan MT (1979) Anatomic models of the tracheobronchial and pulmonary regions of the rat. Anat Rec 195: 483–492. 507403 10.1002/ar.1091950308

[35] Switalla S, Lauenstein L, Prenzler F, Knothe S, Forster C, et al.. (2010) Natural innate cytokine response to immunomodulators and adjuvants in human precision-cut lung slices. Toxicol Appl Pharmacol. 10.1016/j.taap.2010.04.010 20434477

[36] Kasper M, Seidel D, Knels L, Morishima N, Neisser A, et al (2004) Early signs of lung fibrosis after in vitro treatment of rat lung slices with CdCl2 and TGF-beta1. Histochem Cell Biol 121: 131–140. 14752665 10.1007/s00418-003-0612-6

[37] Behrsing HP, Furniss MJ, Davis M, Tomaszewski JE, Parchment RE (2013) In vitro exposure of precision-cut lung slices to 2-(4-amino-3-methylphenyl)-5-fluorobenzothiazole lysylamide dihydrochloride (NSC 710305, Phortress) increases inflammatory cytokine content and tissue damage. Toxicol Sci 131: 470–479. 23143926 10.1093/toxsci/kfs319 PMC3551430

[38] Wade L, Bielory L, Rudner S (2012) Ophthalmic antihistamines and H1-H4 receptors. Curr Opin Allergy Clin Immunol 12: 510–516. 22918191 10.1097/ACI.0b013e328357d3ba

[39] Bielory L, Ghafoor S (2005) Histamine receptors and the conjunctiva. Curr Opin Allergy Clin Immunol 5: 437–440. 16131920 10.1097/01.all.0000183113.63311.11

[40] Zampeli E, Tiligada E (2009) The role of histamine H4 receptor in immune and inflammatory disorders. Br J Pharmacol 157: 24–33. 19309354 10.1111/j.1476-5381.2009.00151.x PMC2697784

[41] Gantner F, Sakai K, Tusche MW, Cruikshank WW, Center DM, et al (2002) Histamine h(4) and h(2) receptors control histamine-induced interleukin-16 release from human CD8(+) T cells. J Pharmacol Exp Ther 303: 300–307. 12235264 10.1124/jpet.102.036939

[42] Bongers G, de Esch I, Leurs R (2010) Molecular pharmacology of the four histamine receptors. Adv Exp Med Biol 709: 11–19. 21618883 10.1007/978-1-4419-8056-4_2

[43] Okamoto T, Iwata S, Ohnuma K, Dang NH, Morimoto C (2009) Histamine H1-receptor antagonists with immunomodulating activities: potential use for modulating T helper type 1 (Th1)/Th2 cytokine imbalance and inflammatory responses in allergic diseases. Clin Exp Immunol 157: 27–34. 19659767 10.1111/j.1365-2249.2009.03958.x PMC2710589

[44] Chand N, Eyre P (1975) Classification and biological distribution of histamine receptor sub-types. Agents Actions 5: 277–295. 1979 10.1007/BF02205232

[45] Eyre P, Chand N (1982) Histamine receptor mechanisms of the lung. In: Ganellin CR, Parsons ME, editors. Pharmacology of histamine receptors. Bristol; Boston: PSG. pp. 298–332.

[46] Yre P (1969) The pharmacology of sheep tracheobronchial muscle: a relaxant effect of histamine on the isolated bronchi. Br J Pharmacol 36: 409–417. 4389280 10.1111/j.1476-5381.1969.tb07997.x PMC1703602

[47] Chand N (1980) Distribution and classification of airway histamine receptors: the physiological significance of histamine H2-receptors. Adv Pharmacol Chemother 17: 103–131. 7004138 10.1016/s1054-3589(08)60008-3

[48] Chand N (1980) Is airway hyperactivity in asthma due to histamine H2-receptor deficiency? Med Hypotheses 6: 1105–1112. 7453589 10.1016/0306-9877(80)90131-0

[49] Foreman JC (1991) Histamine H2 Receptors and Lung Function. In: Arrang JM, Uvnäs B, editors. Histamine and histamine antagonists. Berlin; New York: Springer Berlin Heidelberg. pp. 285–304.

[50] Van der Velden J, Barker D, Barcham G, Koumoundouros E, Snibson K (2012) Increased mast cell density and airway responses to allergic and non-allergic stimuli in a sheep model of chronic asthma. PLoS One 7: e37161. 22606346 10.1371/journal.pone.0037161 PMC3351402

